# Hydraulic jumps with low inflow Froude numbers: air–water surface patterns and transverse distributions of two-phase flow properties

**DOI:** 10.1007/s10652-022-09854-5

**Published:** 2022-04-27

**Authors:** Davide Wüthrich, Rui Shi, Hubert Chanson

**Affiliations:** 1 Department of Hydraulic Engineering, Delft University of Technology, 2628 CN Delft, The Netherlands; 2 School of Civil Engineering, The University of Queensland, Brisbane, QLD 4072 Australia

**Keywords:** Hydraulic jump, Low inflow Froude numbers, Energy dissipation, Air–water flow properties, Sidewall effects, Physical modelling, Hydraulic structures, Sidewall optical techniques, Phase-detection probe

## Abstract

**Abstract:**

Hydraulic jumps are commonly employed as energy dissipators to guarantee long-term operation of hydraulic structures. A comprehensive and in-depth understanding of their main features is therefore fundamental. In this context, the current study focused on hydraulic jumps with low Froude numbers, *i.e.* Fr_1_ = 2.1 and 2.4, at relatively high Reynolds number: Re ~2 × 10^5^. Experimental tests employed a combination of dual-tip phase-detection probes and ultra-high-speed video camera to provide a comprehensive characterisation of the main air-water flow properties of the hydraulic jump, including surface flow features, void fraction, bubble count rate and interfacial velocities. The current research also focused on the transverse distributions of air-water flow properties, *i.e.* across the channel width, with the results revealing lower values of void fraction and bubble count rate next to the sidewalls compared to the channel centreline data. Such a spatial variability in the transverse direction questions whether data near the side walls may be truly representative of the behaviour in the bulk of the flow, raising the issue of sidewall effects in image-based techniques. Overall, these findings provide new information to both researchers and practitioners for a better understanding of the physical processes inside the hydraulic jump with low Froude numbers, leading to an optimised design of hydraulic structures.

**Article Highlights:**

- Experimental investigation of air-water flow properties in hydraulic jumps with low Froude numbers
- Detailed description of the main air-water surface features on the breaking roller
- Transversal distribution of the air-water flow properties across the channel width and comparison between centreline and sidewall.

## Introduction

**Fig. 1:**
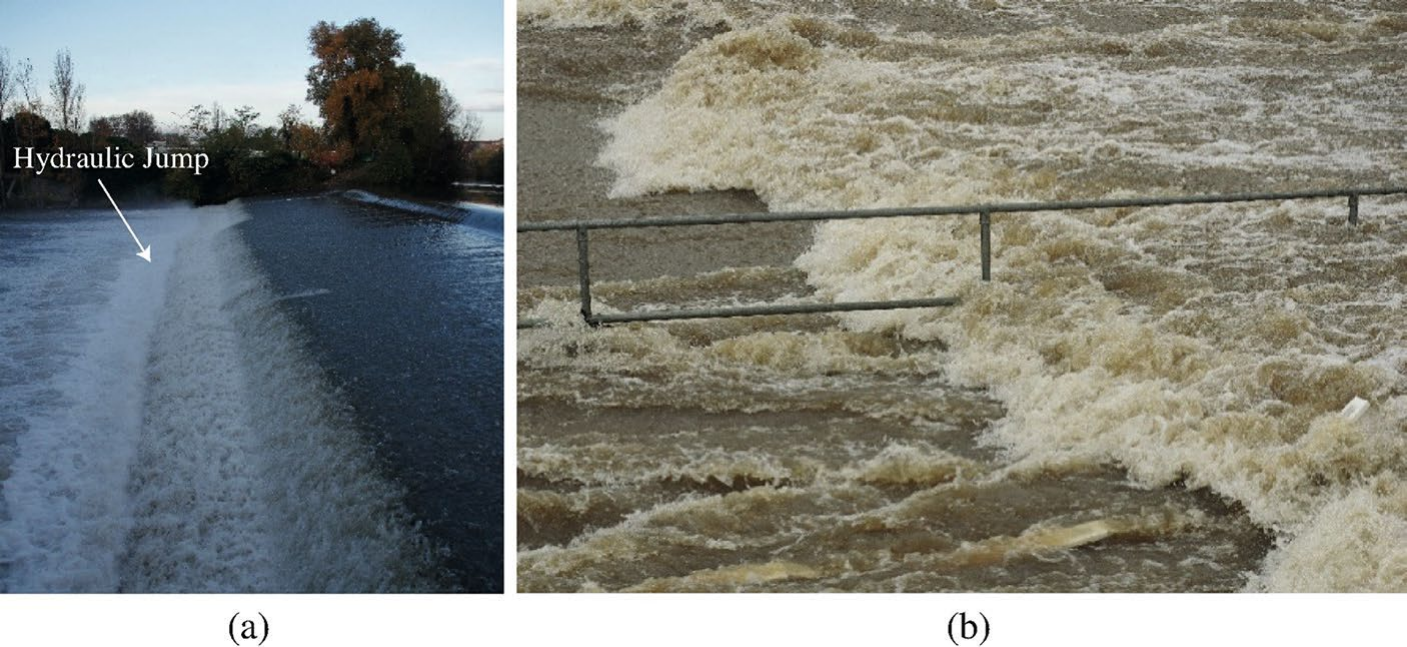
Photographs of hydraulic jumps: **a** Hydraulic jump at the toe of a weir along a branch of the Garonne River in Toulouse (France) on 24 November 2016 [Flow direction from right to left]; **b** Hydraulic jump with a marked roller along Norman Creek, Brisbane (Australia) in a culvert inlet on 30 March 2017 [Flow direction from left to right]

A hydraulic jump is a steady open channel flow characterised by the sudden transition from a supercritical to a sub-critical flow motion [1, 2] (Fig. 1), representing a hydrodynamic singularity [3]. Seminal historical contributions encompassed the experiments of Bidone [4] and Darcy and Bazin [5], and the theoretical developments of Bélanger [6] and Boussinesq [7]. Recent technical reviews included [8–10]. Hydraulic jumps are traditionally used in hydraulic structures to dissipate the kinetic energy of the flow [11–13]. The behaviour of a hydraulic jump is strongly linked to its inflow Froude and Reynolds numbers, both defined as functions of the inflow depth *d*_1_ and velocity *V*_1_:

1 $$ Fr_{1} = \frac{{V_{1} }}{{\sqrt {gd_{1} } }} $$

2 $$ {\text{Re}} = \frac{{\rho V_{1} d_{1} }}{\mu } $$

where *g* is the gravitational constant (*g* = 9.794 m/s^2^ in Brisbane, Australia), *ρ* is the water density, and *μ* is the dynamic viscosity of water. For 1 < Fr_1_ < 1.4, an undular jump is observed, whereas for Fr_1_ > 1.5 to 3.0, a breaking roller may occur [10, 14, 15]. Hydraulic jumps with a marked roller are characterised by some strong three-dimensional motion, intense surface fluctuations, water splashes and substantial air entrainment [8, 11, 16, 17] (Fig. 1). For energy dissipation applications, steady jumps with inflow Froude numbers between 4.5 and 9 provide optimum energy dissipation conditions [2].

While there have been seminal investigations of undular hydraulic jumps [15, 18–20], detailed studies of hydraulic jumps with marked roller are rarer because the presence of air bubbles and gas–liquid interfaces adversely affects the usage of traditional monophase flow instruments, including Prandtl-Pitot tube, LDA, PIV and ADV, unless working at very low Reynolds numbers in absence of air entrainment. Recently, Optical Flow (OF) techniques have been introduced to investigate the air–water flow properties through side-view high-speed videos based on changes in brightness intensity [21–24], although the sidewall data might not be truly representative of the centreline air–water flow properties. Most studies treated the flow as two-dimensional, although Wang and Chanson [25] showed the existence of three-dimensional turbulent structures, suggesting a spatial variation of the air–water flow properties across the roller width. Previous studies mostly focused on hydraulic jumps at relatively large Froude numbers (Fr_1_ > 3), while little attention was given to hydraulic jumps with marked breaking rollers at relatively low Froude numbers, with only a few studies investigating their air–water flow features, as detailed in Table 1. The latter group included the contribution of Murzyn et al. [26], Chachereau and Chanson [27, 28] who conducted both visual observations and air–water flow measurements in breaking hydraulic jumps. To date, the hydrodynamic properties of hydraulic jumps with low Froude numbers remain mostly un-explored, and it is the aim of this contribution to: (1) provide a comprehensive characterisation of the surface features and main air–water flow properties in hydraulic jump with low Froude numbers; (2) discuss their transverse variations across the roller breadth.

**Table 1:** Air–water flow measurements in hydraulic jumps with marked roller at low Froude number (Fr_1_ < 3.0)

	Fr_1_ [-]	*d*_1_ [m]	*V*_1_ [m/s]	*Q* [m^3^/s]	*x*_toe_ [m]	Re [-]	*W* [m]	Instrumentation
Murzyn et al. [26]	2.0	0.059	1.50	0.022	0.36	8.85 × 10^4^	0.3	Optical fibre phase-detection probe
	2.4	0.046	1.64	0.022	0.28	7.54 × 10^4^		
Lennon and Hill [19]	1.4	0.031	0.76	0.006	0.31	7.90 × 10^4^	0.3	Particle Image Velocimetry (PIV)
	1.6	0.031	0.91	0.006	0.15	9.27 × 10^4^		
	3.0	0.020	1.31	0.006	0.26	9.15 × 10^4^		
Valle and Pastermack [57]	2.8	0.216	4.06	–^(1)^	–^(1)^	8.34 × 10^5^	–^(1)^	Two-rod Campbell-Scientific CS615probe
Chachereau and Chanson [27, 28]	2.4	0.042	1.57	0.033	1.50	6.60 × 10^4^	0.5	Acoustic displacement meters & Dual-tip phase detection probe
	2.7	0.042	1.71	0.036	1.50	7.30 × 10^4^		
	2.8	0.044	1.82	0.040	1.50	8.00 × 10^4^		
	2.9	0.045	1.96	0.045	1.50	8.90 × 10^4^		
Wang [58]	2.8	0.029	1.50	0.022	1.50	4.30 × 10^4^	0.5	Video camera, Acoustic displacement meters, & Dual-tip phase detection probe
	3.0	0.024	1.44	0.017	1.50	3.54 × 10^4^		
Montano and Felder [53]	2.1	0.154	2.60	0.200	–^(2)^	4.00 × 10^5^	0.5	Dual-tip phase detection probe
Wüthrich et al. [29]	2.4	0.084	2.21	0.099	1.30	1.86 × 10^5^	0.5	Dual-tip phase detection probe
Estrella et al. [35]	1.9 to 2.1	0.012 to 0.130	1.40 to 2.36	0.004 to 0.153	0.19 to 1.50	7.75 × 10^3^ to 3.05 × 10^5^	0.5	Dual-tip phase detection probe
Present study	2.1	0.097	2.10	0.102	1.50	2.03 × 10^5^	0.5	Dual-tip phase detection probe & Ultra-high-speed video camera
	2.4	0.084	2.21	0.092	1.30	1.86 × 10^5^		

^(1)^Field observations in the upper South Fork American River basin, Sierra Nevada, California; ^(2)^ Classic Hydraulic Jump (CHJ) downstream of a sloped channel; (–): data not available

## Experimental set-up and flow conditions

### Experimental facility

**Fig. 2:**
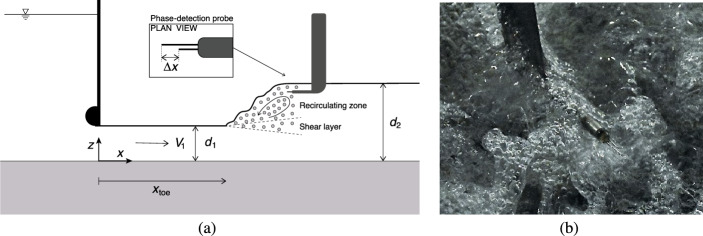
Definition sketch of the main parameters with details of the phase detection probe; **a** side view sketch of the experimental setup; **b** High-shutter speed photograph of the dual-tip phase detection conductivity probe in the hydraulic jump for Fr_1_ = 2.4

All physical tests were conducted in a large-size experimental facility at the University of Queensland, in Brisbane, Australia. The hydraulic jump was generated in a 3.2 m long, 0.5 m wide and 0.4 m deep horizontal rectangular test section. The water was initially conveyed into an upstream head tank equipped with two rows of flow straighteners and a rounded undershoot gate (*Ø* = 0.3 m, Fig. 2a), inducing a horizontal and contraction-less impinging flow in the downstream test section. The latter was built with a smooth HDPE bed and glass sidewalls. The position of the roller toe was constrained at *x*_toe_/*d*_1_ = 15.5 through an adjustable overshoot vertical gate located at the downstream end of the test section, where *x*_toe_ is the roller toe distance from the upstream gate and *d*_1_ is the inflow depth (Fig. 2a). In Fig. 2a, *x* represents the longitudinal coordinate with *x* = 0 at the upstream undershoot gate, while *y* and *z* are the transverse and vertical coordinates, respectively. The same experimental facility was used in several previous studies, including Wang [58], Wang and Chanson [25] and Wüthrich et al. [29].

### Instrumentation

The water discharge was measured using a Venturi flowmeter installed in the supply pipeline with a precision of ± 2%. Both the upstream and downstream water depths were measured with a point-gauge with an accuracy of ± 0.5 mm. The air–water properties in the turbulent roller were investigated using phase-detection dual-tip conductivity probes designed at the University of Queensland (Fig. 2b). Both tips had an inner diameter *Ø* = 0.25 mm and outer diameter *Ø* = 0.8 mm with a transverse separation distance Δ*y* = 1.8 mm (Fig. 2b). For the present study, the longitudinal separation distance between leading and trailing tips Δ*x* ranged between 5.1 and 7.0 mm. The position of the probe in the vertical direction was controlled using a digital ruler with an error of less than ± 0.2 mm. Its position in the transverse direction was measured using a ruler, with a precision of ± 0.5 mm. Based upon previous studies, both probe tips were sampled simultaneously with an acquisition frequency of 20 kHz for a duration of 45 s [30, 58].

A Phantom ultra-high-speed video cameras (v2012) was used to characterise the free-surface features of the hydraulic jump, recording up to 22,700 monochrome frames per second (fps) in full High Definition HD (1280 × 800 pixels). The video camera was installed on the top of the channel to investigate the roller’s upper surface, at a distance of 1.3 m above the free-surface of the incoming supercritical flow. This elevation was sufficient to capture the whole channel width and prevent any droplet to reach the camera lens. Herein, a total of 25 movies were recorded at 22,000 fps in high definition (1280 × 800 pixels) for a duration of 2.25 s each. The camera was equipped with a lens Nikkor™ AF 50 mm *f*1.4, guaranteeing a minimal level of distortion.

### Signal processing

**Fig. 3:**
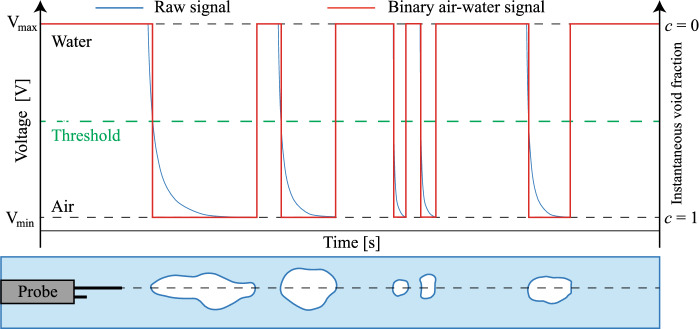
Example of raw measurements and the corresponding air–water signal using a single threshold technique set to 50% of the voltage difference between air and water

Within the air–water flow, the needle-shaped sensors were able to simultaneously detect the air or water phase based on the different values of the electrical resistance [31]. The raw signal was post-processed using a single threshold technique set at 50% of the voltage difference between air and water. This assigned an instantaneous void fraction value of *c* = 1 for air and of *c* = 0 for water. An example of raw measurements and the corresponding air–water signal is presented in Fig. 3.

From the instantaneous void fraction signal *c*, the time-averaged void fraction *C* was defined as the average time spent in air relative to the total time.

3 $$ C = \frac{1}{N}\mathop \sum \limits_{1}^{N} c $$

The bubble count rate *F* was defined as the number of air bubbles or water droplets per second, calculated as half of the total number of air–water and water–air interfaces divided by the sampling duration. The air–water interfacial velocity *V* was obtained through a cross correlation technique as

4 $$ V = \frac{\Delta x}{T} $$

with Δ*x* being the longitudinal distance between the two tips and *T* the time lag corresponding to the maximum cross-correlation coefficient between leading and trailing tip signals [32].

Two bubbles that are closer than a particular time (or length) scale, can be considered part of a cluster. This characteristic water scale may be related to the water chord statistics based on the near-wake criterion [33]. Detailed analysis in terms of bubble clustering provided some further information on the turbulent characteristics across bubbles with different length scales. Herein two bubbles were considered part of a cluster when the water chord time between two consecutive bubbles was less than the bubble chord time of the lead particle:

5 $$ \left( {t_{{{\text{ch}}}} } \right)_{{{\text{water}}}} < \left( {t_{{{\text{ch}}}} } \right)_{{{\text{air}}}} $$

where (*t*_ch_)_water_ is the water chord time between two bubbles, (*t*_ch_)_air_ is the air chord time of the leading bubble. The same approach was used by Chachereau and Chanson [27, 58] in hydraulic jumps. Herein the data was only analysed in the streamwise direction. In relation to clusters, a parameter that was derived from the air–water signal is the cluster count rate *F*_clu_, defined as the average number of clusters per unit time.

### Flow conditions

The physical properties of hydraulic jumps are highly linked to the Froude and Reynolds numbers associated with the incoming flow. Two main test series were conducted in the present study for Fr_1_ = 2.1 and 2.4, and all flow conditions and measurement locations are summarised in Table 2. The tests were conducted with relatively high values of the Reynolds number, *i.e.* Re ~ 2 × 10^5^, thus minimising potential scale effects in air–water flows under a combined Froude and Morton similitude [34, 35]. Herein, the jump toe was located at a longitudinal position *x*_1_/*d*_1_ ~ 15 for Fr_1_ = 2.1 and 2.4, with previous velocity measurements in the same flume showing that, at these jump toe locations, the inflow was characterised by a partially developed boundary layer [27, 28, 58].

**Table 2:** Summary of the hydraulic jump flow conditions for air–water flow measurements in the present study

Fr_1_	Re	*d*_1_ [m]	*V*_1_ [m/s]	*Q* [m^3^/s]	*x*_toe_ [m]	(*x*-*x*_toe_)/*d*_1_	Centreline data	Sidewall data	Transverse data
							*y*/*W*	*y*/*W*	*z*/*d*_1_
2.1	2.03 × 10^5^	0.097	2.10	0.102	1.50	0.35	0.5	–	–
						0.60	0.5	0.024	–
						0.89	0.5	–	–
						1.04	0.5	–	–
						1.19	0.5	0.024	1.14
						1.48	0.5	–	–
						2.38	0.5	–	–
						3.57	0.5	0.024	1.37
						4.76	0.5	–	–
2.4	1.86 × 10^5^	0.084	2.21	0.092	1.30	0.60	0.5	–	–
						1.19	0.5	0.024	0.95, 1.01, 1.07, 1.19, 1.37, 1.43
						2.38	0.5	–	–
						3.57	0.5	0.024	1.01, 1.19, 1.37, 1.90
								0.012	
								0.005	
						4.76	0.5	–	–

Fr_1_ = Froude number, *d*_1_ = inflow depth, *V*_1_ = inflow velocity, *Q* = discharge, *x*_toe_ = position of the roller toe, Re = Reynolds number, *W* = channel width (*W* = 0.5 m herein), *x* = horizontal direction, *y* = transversal direction, *z* = vertical direction

## Visual observations

**Fig. 4:**
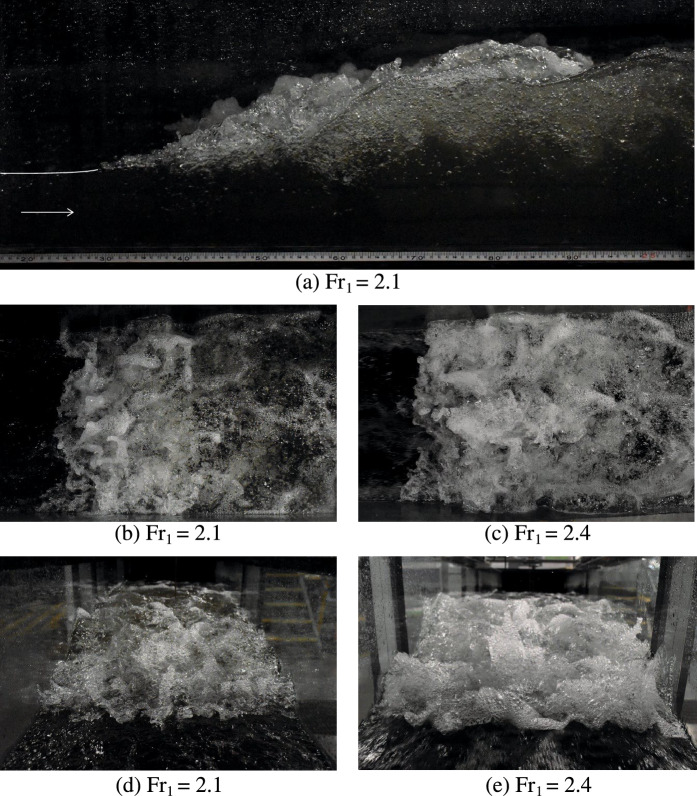
Visual observations of hydraulic jumps with Fr_1_ = 2.1 and 2.4

The present study focused on hydraulic jumps with a breaking roller at low Froude numbers (Fr_1_ = 2.1 and 2.4). Typical flow patterns are illustrated in Fig. 4. Both flow conditions revealed the presence of a breaking roller with a fluctuating behaviour and relatively intense air entrainment, considering the low inflow Froude numbers. Several studies suggested that the turbulent fluid motion could result in a deformation of the free surface, leading to enhanced surface roughness, breakup, and disintegration [36–39]. Figure 4d, e show the sudden increase in water depth at the roller toe, with the formation of three-dimensional "*foamy*" structures, characterised by substantial air entrainment and entrapment. The breaking roller showed classic air–water flow features, including a well-defined shear layer and a recirculation region above, as previously seen in hydraulic jumps with higher Froude numbers [40, 41, 58].

The roller length *L*_r_ was estimated as the distance over which the mean free surface level increased monotonically, following Murzyn and Chanson [56] and Wang [58]. In the present study, the roller length was estimated to be *L*_r_ ~ 0.65 m (*L*_r_/*d*_1_ ~ 8) for Fr_1_ = 2.4 and *L*_r_ ~ 0.63 m (*L*_r_/*d*_1_ ~ 6.5) for Fr_1_ = 2.1. Despite the uncertainties liked to the fluctuating and oscillating behaviour of the jump (*i.e.* ± 1 or 2 cm), these measurements were in agreement with the findings of Wang [58] and Wang and Chanson [42] for 2 < Fr_1_ < 10:

6 $$ \frac{{L_{r} }}{{d_{1} }} = 6 \cdot \left( {Fr_{1} - 1} \right) $$

### Air–water surface features

**Fig. 5:**
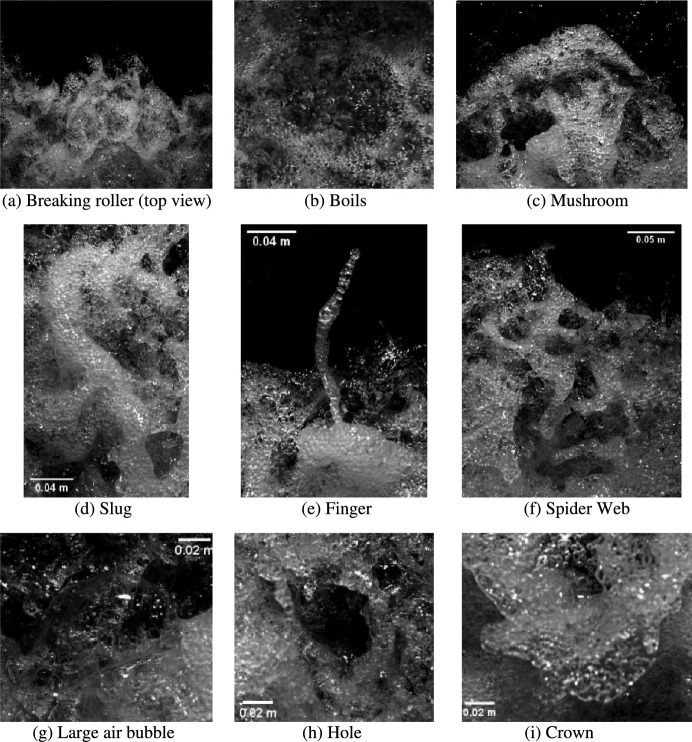
Top view of the strong free surface turbulence observed in a breaking roller with Reynolds number Re = 1.86 × 10^5^ and Froude number Fr_1_ = 2.4: **a** Top view of the roller; **b** Boil; **c** Mushroom; **d** Slug; **e** Finger; **f** Spider web; **g** Large air bubble; **h** Hole; **i** Crown. Initial flow direction from top to bottom

All hydraulic jumps presented a marked breaking process with a highly turbulent roller and a sizeable amount of air entrainment and entrapment. The flow motion at the roller toe (top view) was investigated for a hydraulic jump with Re = 1.86 × 10^5^ and Fr_1_ = 2.4 by means of the ultra-high-speed video camera installed on top of the channel. The video movies captured a number of key air–water surface features, which complement the observations of Wüthrich et al. [39] for non-stationary breaking bores. Examples of these air–water flow surface motions are presented in Fig. 5, revealing the complexity of their geometry and the interactions that occurred between these features.

The chaotic behaviour of the air–water mixture generated short-lived, three-dimensional, air–water surface features that were the result of a complex turbulent motion strongly linked to the physical processes occurring within the roller. The duration (*i.e.* lifespan) of these features was smaller than a second, thus making ultra-high-speed video data a basic requirement for a comprehensive assessment. Video analysis showed that during their lifespans, these air–water surface features evolved in both space and time, interacting with each other before disappearing within the roller, often without generating splashes. Within all these features, several reoccurring surface characteristics were identified. In hydraulic jumps, such air–water surface features included fingers, water droplets, crowns, slugs, spider webs, mushrooms, boils and holes. It was noted that most features were also previously observed in a breaking bore, which was considered a hydraulic jump in transition [39]. It is important to point out that the focus of the high-speed video camera only captured the first half of the roller, thus explaining why crowns and boils were not observed for hydraulic jumps, as these features were more common in the downstream part of the bore’s breaking roller.

**Fingers** were elongated mono-axial ejections in which the length was significantly greater than its width. These features were the result of a mostly upward ejection of an air–water volume, occurring predominantly in the first half of the roller, close to roller toe. The existence of these features was previously reported by Murzyn and Chanson [56], Chanson [43], Chachereau and Chanson [28] for hydraulic jumps with higher Froude numbers and Wüthrich et al. [39] for breaking bores. Whilst, in breaking bores, the direction of the fingers was mostly pointing against the propagation of the bore roller, both directions were commonly observed in hydraulic jumps (Fig. 5). Fingers were made of smaller bubbles, with a single bubble occasionally occupying the whole finger width (Fig. 5e). During their lifespan, fingers showed the appearance of Plateau-Rayleigh instabilities, partially responsible for their breakage into smaller droplets of smaller total surface area [44]. These **water droplets** were often projected upstream of the roller toe, as reported by Leng and Chanson [55] and Wüthrich et al. [39].

**Slugs** are defined as *S*-shaped ribbons of foamy mixture, primarily aligned parallel to the direction of the flow. Slugs were foamy entities characterised by a very high local concentration of air bubbles, resulting into locally higher void fractions. As visualised in Fig. 5d, the shape of the slugs and the amount of air bubbles in the feature were visually similar to those identified in breaking bores. **Holes** were three-dimensional and short-lived air cavities within the roller surface, surrounded by other air–water features (Fig. 5h). These appeared for all tested Froude numbers and were characterised by a darker colour, *i.e.* clear water beneath the air cavity. Holes were associated with mostly circular shapes, with a length comparable to the width, in line with previous findings in breaking bores. **Spider webs** were very-short-lived features, resulting from the complex interaction between multiple features including fingers, ejections and other foamy structures (Fig. 5f). This complex connectivity resulted into a mesh of thin structures and holes, assuming the form of a spider web. Because of their short duration, the shape of these features evolved very quickly, leading to a jagged or indented profile of the roller toe perimeter. Their rapid dissapearance within the incoming flow was sometimes responsible for the local backward motion of the roller toe. Often these features followed the ejections of a **mushroom**, which consisted of an accumulation of pseudo-circular foamy mixture of air and bubbles towards the roller toe with a pseudo semi-circular shape (Fig. 5c). All these features were previously observed in breaking bores, thus confirming similarities between stationnary and non-stationary flows.

Additional air–water flow features commonly observed on the surface of breaking bores were crowns and boils. **Crowns** were surface features generated by air–water ejections with a pseudo-circular shape, where its length was smaller than its width. **Boils** were annular patterns resulting from an upward flow motion reaching the free surface from within the roller. Both features typically occurred in the second half of the roller and were scarcely observed herein, where the focus was the first half of the roller.

**Fig. 6:**
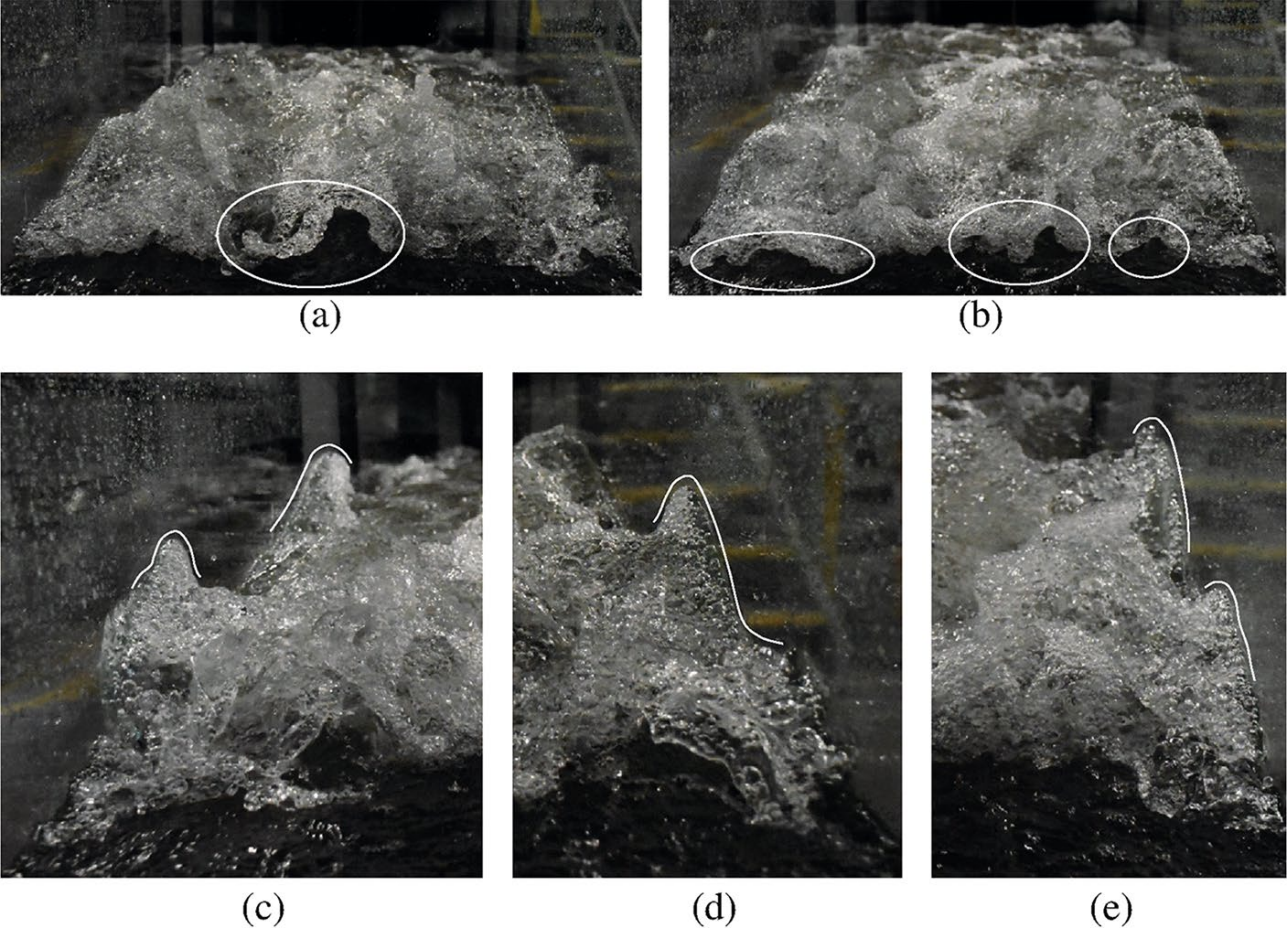
Photographs of caves (**a**, **b**) and vertical structures along the sidewall (**c**, **d** and **e**) for a hydraulic jump with Fr_1_ = 2.1 (*d*_1_ = 0.097 m, *V*_1_ = 2.1 m/s, Re = 2.03 × 10^5^). Initial flow from foreground to background. Shutter speed 1/800 s

Close observations also revealed the presence of several large air bubbles with a thin film (Fig. 5g). These features were more common for lower Froude numbers and could reach diameters of ~ 100 mm before breaking. The rupture of the film often occurred form the centre of the dome and quickly propagated towards the outer part of the bubble. In addition, some images were obtained with dSLR photography for the hydraulic jump with Fr_1_ = 2.1 from the upstream side. Figure 6a, b revealing the presence of air cavities located below the roller, similar to “*caves*”, as previously indicated by Chachereau and Chanson [28]. These features were believed to contribute to air entrapment in the roller and the overall aeration of the breaking hydraulic jump. These cavities were short-lived features, and some examples are presented in Fig. 6a, b for Fr_1_ = 2.1. Some additional features were observed attached to the sidewalls, characterised by some vertical flow motion (Fig. 6c, d and e). These were believed to be the result of the presence of the sidewalls, affecting and disrupting the air–water flow motion in the transverse direction. These features might explain some differences in air–water flow properties recorded between the sidewall and the centreline, further discussed in Sect. 5.

## Air–water flow properties

This section focuses on the air–water flow properties of hydraulic jumps on the channel centreline with low Froude numbers and high Reynolds numbers. The experimental flow conditions for Fr_1_ = 2.1 and 2.4 are summarised in Table 2. For each flow condition, the air water flow properties were analysed in terms of void fraction (Sect. 5.1), bubble count rate (Sect. 5.2), bubble/droplet chord time and clustering (Sect. 5.3) and interfacial velocities (Sect. 5.4). The air–water flow properties for both Froude numbers were systematically measured both at the centreline (*y*/*W* = 0.5) and next to the sidewall (*y*/*W* < 0.024), where *W* is the channel width (*W* = 0.5 m). The air–water flow properties near the sidewalls and across the channel width are further discussed in Sect. 5.

### Void fraction

**Fig. 7:**
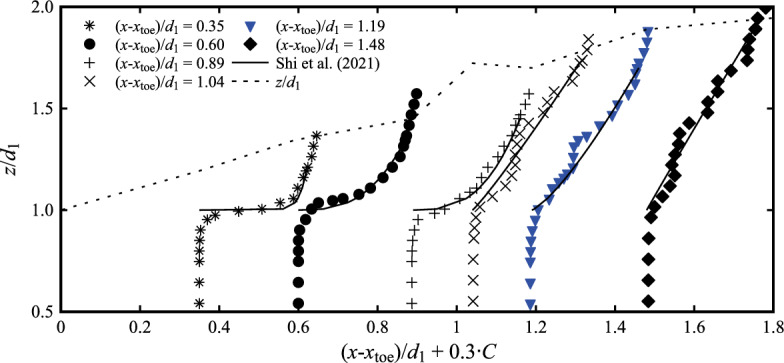
Vertical profiles of the void fraction *C* near the roller toe for Fr_1_ = 2.1 [Flow conditions: Fr_1_ = 2.1, *d*_1_ = 0.097, *V*_1_ = 2.10 m/s] - Channel centreline data

Previous studies showed that hydraulic jumps with a marked roller exhibited two regions with different behaviours: (1) a lower shear layer region and (2) a recirculating region in the upper part of the roller [26, 40, 45, 58]. The shear layer developed in the lower part of the roller, generating a convective transport of air bubbles entrapped at the hydraulic jump toe and advected downstream [46]. The present data revealed that, close to the roller toe, the jump did not present a fully developed shear layer, suggesting the existence of a different diffusion process. The lack of a peak in void fraction in the shear layer region for (*x*-*x*_toe_)/*d*_1_ < 1.0 was also observed in the data by Chachereau and Chanson [27] for Fr_1_ = 3.1. Investigations in the region 0 < (*x*-*x*_toe_)/*d*_1_ < 1.0 showed a behaviour characterised by a monotonically increasing curve, as shown in Fig. 7. In that region, the void fraction *C* was best described by the advection–diffusion model developed by Shi et al. [23] for highly unsteady flows in the form:

7 $$ C = 0.9\left( {\frac{{z - d_{1} }}{{Z_{90} - d_{1} }}} \right)^{N}\quad \text{valid}\quad\text{for}\quad 0 < \frac{{x - x_{{{\text{toe}}}} }}{{d_{1} }} < 1 $$

where *Z*_90_ is the elevation where *C* = 0.9 and *N* an empirical coefficient defined as a function of the depth-averaged void fraction in the roller $C_{{{\text{mean}}}}^{*}$

8 $$ C_{{{\text{mean}}}}^{*} = \frac{1}{{Z_{90} - d_{1} }}\mathop \smallint \limits_{{d_{1} }}^{{Z_{90} }} C\,\text{d}z = \frac{0.9}{{N + 1}} $$

Typical results are presented in Fig. 7 for the locations close to the roller toe, showing an excellent agreement between Eq. (7) and data for (*x*-*x*_toe_)/*d*_1_ < 1, where a local void fraction peak in the shear layer was not observed. Altogether, the present data confirmed the finding of Estrella et al. [35], showing a convex void fraction profile near the toe.

**Fig. 8:**
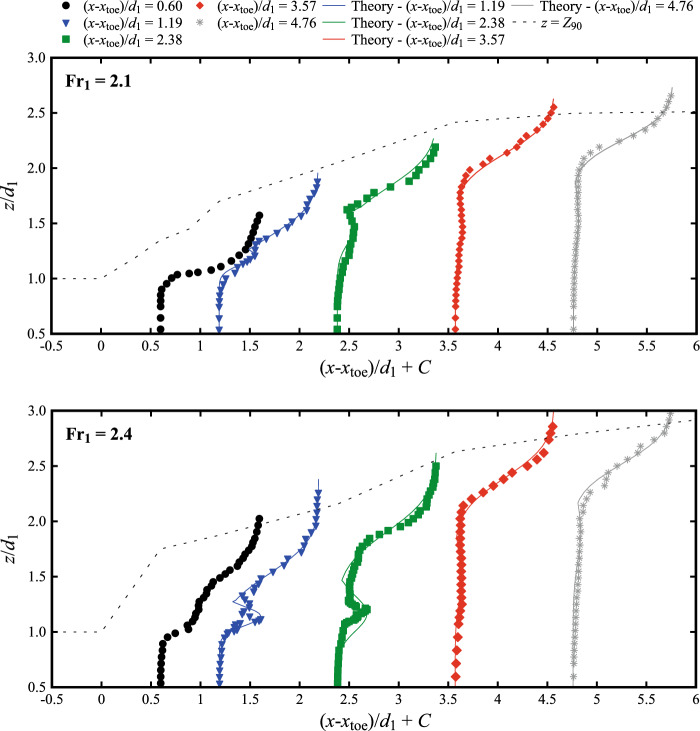
Void fraction profiles at different longitudinal locations along the centreline with: (top) Fr_1_ = 2.1, *d*_1_ = 0.097, *V*_1_ = 2.10 m/s (bottom) Fr_1_ = 2.4, *d*_1_ = 0.084, *V*_1_ = 2.21 m/s. Channel centreline data. Theory refers to the analytical solution of the advection–diffusion equation [40]. Legend applies to both figures

Further away from the roller toe [(*x*-*x*_toe_)/*d*_1_ > 1.0], typical void-fraction profiles for both Froude numbers Fr_1_ = 2.1 and 2.4 are presented in Fig. 8 at (*x*-*x*_toe_)/d_1_ = 0.60, 1.19, 2.38, 3.57 and 4.76. The data are also compared to the characteristic elevation *Z*_90_ where the void fraction is *C* = 0.90. With respect to hydraulic jumps with higher Froude numbers, the present data showed some differences in the turbulent shear layer, with a small local peak in void fraction, only observed for 1.19 < (*x*-*x*_toe_)/*d*_1_ < 2.38, becoming almost undetectable for Fr_1_ = 2.1 (Fig. 7). At larger distances from the roller toe, no peak in void fraction was observed, showing a more uniform void fraction profile throughout the lower roller region. In the recirculating region, lesser differences were observed with stronger hydraulic jumps.

For 1.19 < (*x*-*x*_toe_)/*d*_1_ < 2.38, the void fraction data showed a good agreement with the analytical solution of the advection–diffusion equation for air bubbles [26, 40, 47, 48]. These analytical solutions include dimensionless diffusivity coefficients computed according to the semi-empirical expressions proposed by Wang [58] as functions of the longitudinal position *x*-*x*_toe_ within the roller length *L*_r_, defined in Eq. 6. Although [58] introduced these semi-empirical expressions for 3.8 < Fr_1_ < 10, a good agreement was found with the present experimental data (Fr_1_ = 2.1 and 2.4), thus extending their validity to lower Froude numbers for (*x*-*x*_toe_)/*d*_1_ > 1.

The depth-averaged void fraction *C*_mean_ was estimated as

9 $$ C_{{{\text{mean}}}}^{{}} = \frac{1}{{Z_{90} }}\mathop \smallint \limits_{0}^{{Z_{90} }} C\,\text{d}z $$

**Fig. 9:**
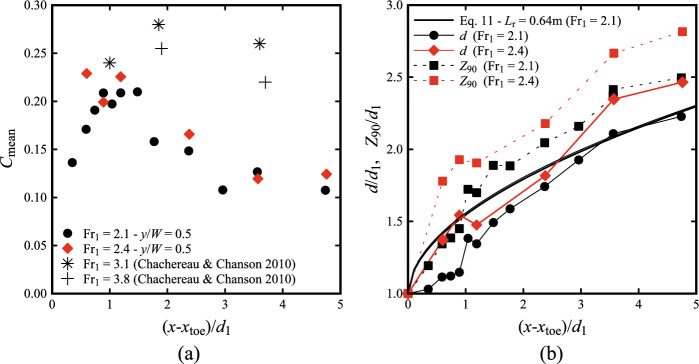
**a** Longitudinal distribution of the mean void fraction *C*_mean_ for Fr_1_ = 2.1 (black symbols), Fr_1_ = 2.4 (red symbols) and comparison with Chachereau and Chanson [27] for Fr_1_ = 3.1 and 3.8; **b** Characteristic elevations *d* and *Z*_90_ for Fr_1_ = 2.1 and 2.4 and comparison with self-similar profile in Eq. (11) [58]. All data refers to the centreline (*y*/*W* = 0.5)

*C*_mean_ describes the rate of air-entrainment in the hydraulic jump, where values of *C* > 0.9 are neglected, as these mostly corresponded to splashing and detached droplets. The results in terms of *C*_mean_ are presented in Fig. 9a as a function of the longitudinal distance from the roller toe, showing a maximum around (*x*-*x*_toe_)/*d*_1_ ~ 1. A decreasing behaviour was observed further downstream for both flow conditions. The lowest Froude number Fr_1_ = 2.1 revealed a lesser depth-averaged void fraction for (*x*-*x*_toe_)/*d*_1_ < 1. Current results were also compared with previous data from Chachereau and Chanson [27] for Fr_1_ = 3.1 and 3.8, revealing the presence of peak value at slightly larger distances from the roller toe (Fig. 9a).

In addition, the equivalent clear water depth *d* was computed at all locations for both Froude numbers

10 $$ d = \mathop \smallint \limits_{0}^{{Z_{90} }} \left( {1 - C} \right)\,\text{d}z = Z_{90} \left( {1 - C_{{{\text{mean}}}} } \right) $$

The longitudinal distribution of *d* is presented in Fig. 9b for both Froude numbers, showing an increasing behaviour downstream of the roller toe. The data was also compared to the self-similar profile expression introduced by Wang [58]:

11 $$ \frac{{d - d_{1} }}{{d_{2} - d_{1} }} = \left( {\frac{{x - x_{{{\text{toe}}}} }}{{L_{r} }}} \right)^{0.537} $$

In summary, the present void fraction results were consistent with those at higher Froude numbers for (*x*-*x*_toe_)/*d*_1_ > 1.

### Bubble count rate

**Fig. 10:**
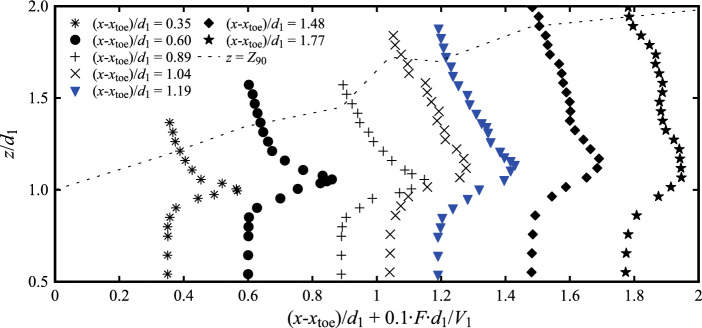
Vertical profiles of the bubble count rate *F* near the roller toe for Fr_1_ = 2.1. Channel centreline data

The bubble count rate *F* represents the average number of water-to-air interfaces detected per second. A strong hydraulic jump typically exhibits a “*bimodal*” vertical profile, with a large peak in the shear layer and a secondary one in the upper recirculating region [10, 40]. However, a closer scrutiny at the behaviour of the bubble count rate at multiple locations in the proximity of the roller toe is presented in Fig. 10, revealing the presence of a single peak for (*x*-*x*_toe_)/*d*_1_ < 1.5. Data showed that the secondary peak in the upper recirculating region started to appear for (*x*-*x*_toe_)/*d*_1_ ≥ 1.77, in line with previous data from [27, 28].

**Fig. 11:**
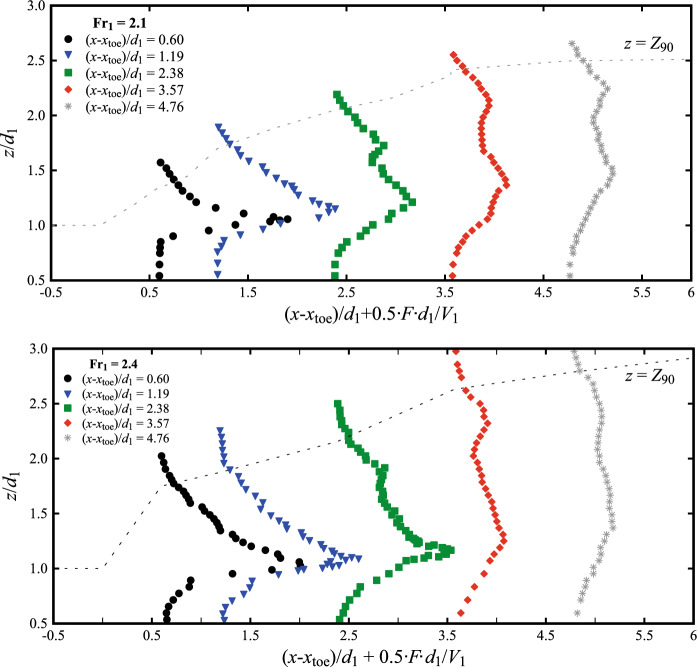
Bubble count rate at different longitudinal locations from the roller toe for: (top) Fr_1_ = 2.1, *d*_1_ = 0.097, *V*_1_ = 2.10 m/s (bottom) Fr_1_ = 2.4, *d*_1_ = 0.084, *V*_1_ = 2.21 m/s. Channel centreline data

Typical profiles obtained at different locations downstream of the roller toe are presented in a normalised form in Fig. 11 for both Fr_1_ = 2.1 and 2.4. Herein, a marked peak in the shear layer was systematically recorded, with the peak becoming less pronounced in the downstream part of the roller due to advection–diffusion processes within the roller.

**Fig. 12:**
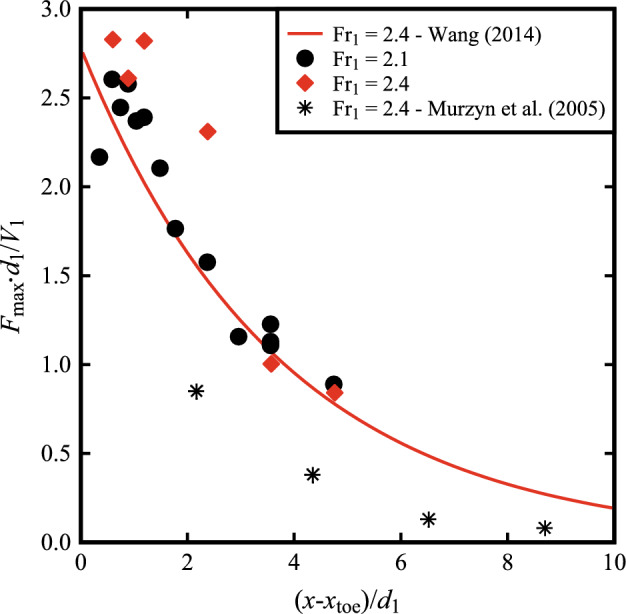
Maximum bubble count rate *F*_max_ as a function of the longitudinal distance from the roller toe for Fr_1_ = 2.1, Fr_1_ = 2.4 and comparison with Murzyn et al. [26] for Fr_1_ = 2.4. and Re = 7.54 × 10^4^. Channel centreline data

The maxima in bubble count rate *F*_max_ in the shear layer are plotted as a function of the longitudinal distance from the roller toe in Fig. 12, where one can notice a power law decay in the shear layer. The values of *F*_max_ were compared to an empirical correlation suggested by Wang [58] for 3.1 < Fr_1_ < 11.2 and 3.5 × 10^4^ < Re < 1.6 × 10^5^. Although not validated for the current flow conditions (Fr_1_ = 2.4 and 2.1), Fig. 12 suggests a good agreement with the experimental data, hinting that Wang's [58] correlation could be extended to lower Froude numbers and higher Reynolds numbers. For Fr_1_ = 2.1, the data showed a maximum in *F*_max_ for (*x*-*x*_toe_)/*d*_1_ ~ 1, with a decreasing behaviour towards the roller toe ((*x*-*x*_toe_)/*d*_1_ → 0). Such a pattern was observed for plunging jets [47] and hinted in hydraulic jumps [58], suggesting that entrained bubbles were broken up into smaller-size bubbles immediately downstream of the entrapment point [49, 54]. Maximum values of bubble count rate *F*_max_ were compared to those previously reported by Murzyn et al. [26] for the same Froude number (Fr_1_ = 2.4) but smaller Reynolds number (Re = 7.54 × 10^4^), showing a larger number of bubbles for the present study. This confirmed the strong link between the maximum bubble count rate and Reynolds number within the shear layer [34, 35]. Note that the data from Murzyn et al. [26] was captured with an optical fibre probe with a diameter *Ø* = 10 μm, whereas the present study employed probes with *Ø* = 0.25 mm. The influence of probe sensor size on the bubble count rate in two-phase flows was discussed for self-aerated stepped chutes [50, 51], showing that smaller sensors can capture a larger number of small bubbles.

### Bubble and droplet chord times distribution

**Fig. 13:**
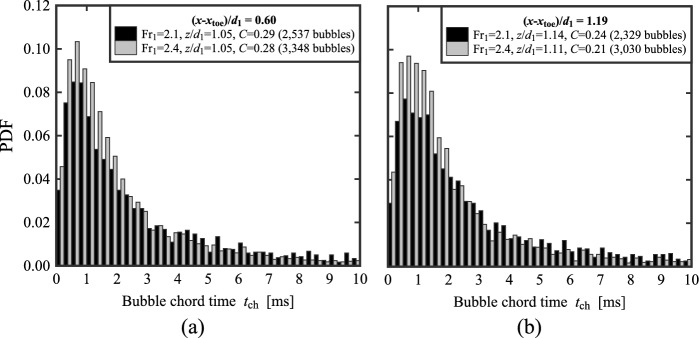
PDF of the bubble chord time at the elevation where *F* = *F*_max_ for both Fr_1_ = 2.1 and 2.4 at two selected locations behind the jump toe (*x*-*x*_toe_)/*d*_1_ = 0.60 and 1.19. Channel centreline data

**Fig. 14:**
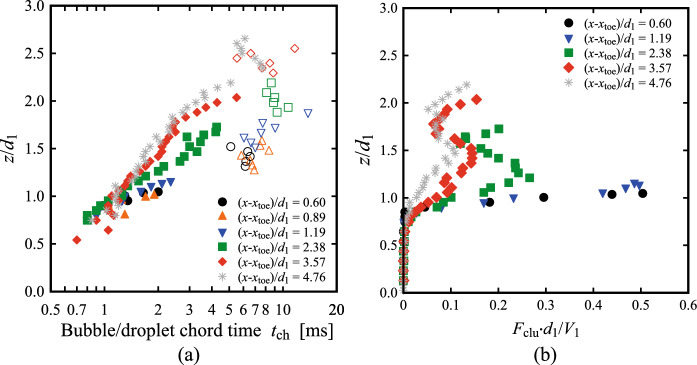
Fr_1_ = 2.1 (*d*_1_ = 0.097 m, *V*_1_ = 2.10 m/s): **a** Vertical distributions of the median particle chord time *t*_ch_; full symbols represent bubble chord times (*C* < 0.3), while empty symbols represent water droplets chord times (*C* > 0.7); **b** Cluster count rate *F*_clu_ for *C* < 0.3 at different locations behind the roller toe. Channel centreline data

For each bubble, its chord time *t*_ch_ was computed as the duration between the water-to-air interface and the subsequent air-to-water interface. Herein the bubble chord times are considered only in the lower part of the flow, where 0.001 < *C* < 0.3. Typical probability distribution functions (PDF) of bubble chord times at the elevation where *F* = *F*_max_ and *C* < 0.3 are presented in Fig. 13 for both Fr_1_ = 2.1 and 2.4 at two selected locations behind the roller toe, *i.e.* (*x*-*x*_toe_)/*d*_1_ = 0.6 and 1.19. Despite the lower number of bubbles detected for Fr_1_ = 2.1 compared to Fr_1_ = 2.4, the data showed relatively similar behaviours for both flow conditions at both locations behind the roller. The shape of the PDFs revealed a maximum at *t*_ch_ ~ 1 ms and a smaller number of small bubbles (*t*_ch_ < 0.5 ms), in line with the results of Chachereau and Chanson [27] and Wang [58] for higher Froude numbers. The data showed highly skewed distributions for all configurations. The data were analysed in terms of median values to limit the effect of extreme values, in line with Wüthrich et al. [52] and Shi et al. [23]. Typical results for bubble chord times for 0.001 < *C* < 0.3 and water droplets chord times for *C* > 0.7 are plotted in Fig. 14a for both Froude numbers, showing an increasing behaviour of bubble chord time for *z*/*d*_1_ > 1 at all locations behind the roller toe. The water droplet data (empty symbols in Fig. 14a) revealed higher scatter and longer chord times compared to air-bubbles.

### Bubble clustering

Entrained bubbles interacted with the turbulent structures of the roller, generating clusters that contributed to the energy dissipation of the flow [9]. In line with previous studies [27, 28, 58], the definition of a cluster was based on a near-wake criterion and two bubbles were considered part of the same cluster when the water chord time between two consecutive bubbles was less than the bubble chord time of the lead particle (Sect. 2.3). Note that herein only the signal from the leading tip was analysed and transversal clustering was not considered. Similarly to the bubble chord time, only data in the region where *C* < 0.3 was considered for bubble clustering properties. From the air–water signal, the cluster count rate *F*_clu_ was defined as the number of clusters per second and the vertical distributions for both Froude numbers are presented in Fig. 14b at multiple locations behind the toe. The data showed a similar trend to the bubble count rate *F*, with vertical profiles closer to roller toe characterised by a single peak in the shear layer region.

### Interfacial velocities

**Fig. 15:**
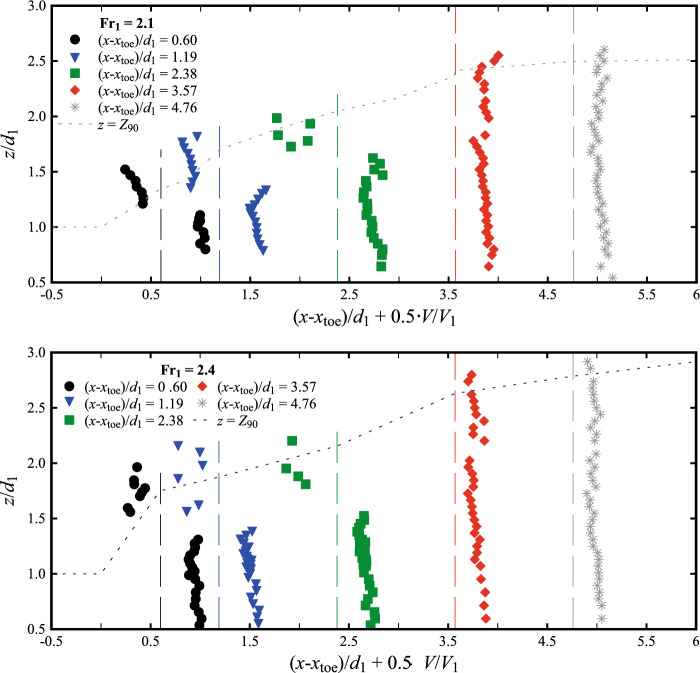
Air–water interfacial velocities at different locations from the roller toe for: (top) Fr_1_ = 2.1, *d*_1_ = 0.097, *V*_1_ = 2.10 m/s; (bottom) Fr_1_ = 2.4, *d*_1_ = 0.084, *V*_1_ = 2.21 m/s. Dashed vertical lines represent reference lines for *V*/*V*_1_ = 0 at each (*x*-*x*_toe_)/*d*_1_ position. Channel centreline data

The longitudinal air–water interfacial velocities were obtained through a cross-correlation analysis between the signals recorded by the leading and trailing tips of the phase-detection probe. Typical data are presented in Fig. 15 at several longitudinal locations downstream of the roller toe. The data showed positive velocity values in the lower part of the flow (*i.e.* the shear layer), whilst the upper recirculating region was characterised by a few negative values close to the roller toe. The elevation of the transition between the two zones increased in the streamwise direction until only positive values were observed for (*x*-*x*_toe_)/*d*_1_ > 3.57. Despite some scatter in the recirculation region and non-aerated part below the shear layer, the results for both Froude numbers showed consistent behaviours with previous studies [25, 26, 40].

## Transversal variations of the air–water flow properties

Recent times have seen an increasing interest in non-intrusive image-based techniques in free-surface flows, *e.g.* Bubble Image Velocimetry (BIV), Particle Image Velocimetry (PIV) and Optical Flow (OF). Thus, a seminal question raises: “*Are the air–water flow properties next to the sidewall representative of the centreline data and of the bulk of the air–water flow*?” While differences between the centreline and sidewall data were mentioned in previous studies [21, 22, 24, 29], a comprehensive data set is missing on the spatial three-dimensional distributions of the main air–water flow properties in hydraulic jumps. Sidewall data at two locations for Fr_1_ = 2.1 and three for Fr_1_ = 2.4 were collected, at *y*/*W* = 0.024 (*i.e.* 12 mm from the sidewall). This distance was chosen as it represented a depth of field, commonly used in image processing techniques. Further, at a selected location [Fr_1_ = 2.4, (*x*-*x*_toe_)/*d*_1_ = 3.57], two additional locations closer to the sidewall (*y*/*W* = 0.012 and 0.005), were tested, *i.e. y* = 6 and 2.5 mm respectively. In addition, some complete transverse profiles were recorded for both Froude numbers Fr_1_ = 2.1 and 2.4 at two longitudinal locations, *i.e.* (*x*-*x*_toe_)/*d*_1_ = 1.19 and 3.57, at select vertical elevations (0.95 ≤ *z*/*d*_1_ ≤ 1.90) within the roller. Complete flow conditions and tested configurations are presented in Table 2.

### Void fraction and bubble count rate

**Fig. 16:**
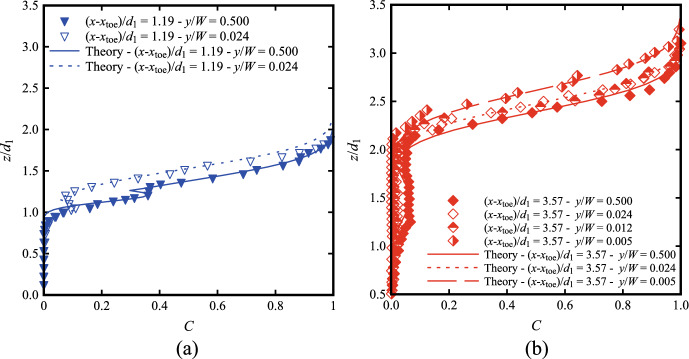
Void fraction profiles at the centreline and near the sidewall at two longitudinal locations from the roller toe for: **a** Fr_1_ = 2.1, (*x*-*x*_toe_)/*d*_1_ = 1.19 and **b** Fr_1_ = 2.4, (*x*-*x*_toe_)/*d*_1_ = 3.57. Theory refers to the analytical solutions of the advection–diffusion equation [40]

For both Froude numbers, the vertical profiles of void fraction were measured next to the sidewall at different longitudinal locations behind the roller toe. Typical profiles are compared to centreline data in Fig. 16 at two locations for Fr_1_ = 2.1 and 2.4, where the results revealed systematically a lesser aeration next to the sidewall, with large differences both in the lower shear layer and upper recirculating zone. In the lower shear layer, the local peak in void fraction was less pronounced next to the sidewall. Table 3 summarises the local maxima of void fractions in the shear layer (*C*_max_) for both Froude numbers, revealing values approximately 2 to 4 times higher in the centreline compared to the sidewalls. In the recirculating zone, data suggested a higher free-surface position near the sidewalls, possibly associated with the vertical motions of the surface-features reported in Fig. 6c, d and e. Similar trends were observed for *y*/*W* = 0.024 and 0.012, with lesser aeration captured at the closest location to the sidewall (*y*/*W* = 0.005). Despite this difference, the data also showed a good agreement between the void fraction profiles and theoretical solutions, both in the centreline and near the sidewall.

**Table 3:** Summary of the values of maximum void fraction in the shear layer *C*_max_ and bubble count rates *F*_max_ measured in the shear layer at the centreline (CL, *y*/*W* = 0.5) and the sidewall (SW, *y*/*W* = 0.024)

Fr_1_	(*x*-*x*_toe_)/*d*_1_	*C*_max,CL_	*C*_max,SW_	*C*_max,CL_*/C*_max,SW_	*F*_max,CL_	*F*_max,SW_	*F*_max,CL_*/F*_max,SW_
2.1	0.60	–^(1)^	–^(1)^	–^(1)^	56.38	26.31	2.14
	1.19	0.360	0.110	3.27	51.76	18.98	2.72
	3.57	0.072	0.016	4.50	23.98	12.89	1.86
2.4	1.19	0.418	0.142	2.94	74.20	20.18	3.68
	3.57	0.067	0.035	1.91	26.40	11.80	2.24

^(1)^No maximum in void fraction was observed in the shear layer (Fig. 8)

**Fig. 17:**
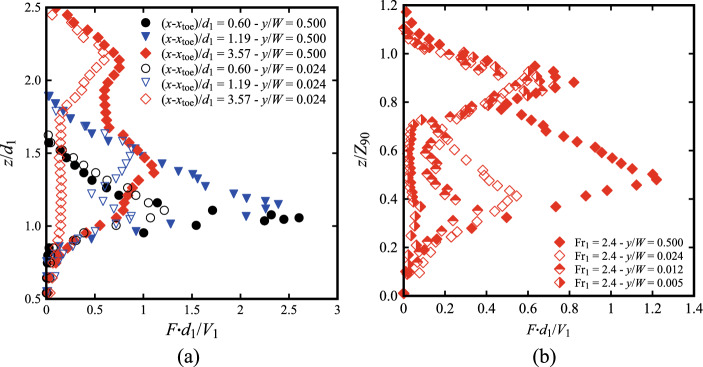
Bubble count rate profiles at the centreline (*y*/*W* = 0.5) and near the sidewall (*y*/*W* < 0.024) at multiple longitudinal locations from the roller toe for: **a** Fr_1_ = 2.4, (*x*-*x*_toe_)/*d*_1_ = 0.60, 1.19 and 3.57; **b** Fr_1_ = 2.4, (*x*-*x*_toe_)/*d*_1_ = 3.57 (4 locations *y*/*W* = 0.5, 0.024, 0.012 and 0.005)

The vertical distributions of bubble count rate were also measured next to the sidewall (*y*/*W* < 0.024) and the data are presented for Fr_1_ = 2.4 in Fig. 17. A lower number of bubbles was systematically detected next to the sidewalls, compared to the centreline data. Little difference was observed in the upper part of the roller, where the process was dominated by the fluctuations of the free-surface (*z*/*Z*_90_ > 0.8 in Fig. 17b). However, both graphs in Fig. 17 show significant differences in the shear layer region for both Froude numbers. The maximum values summarised in Table 3 show that peaks in bubble count rate were approximately 2 to 4 times smaller in the vicinity of the sidewall, as earlier reported by Wüthrich et al. [29] for similar flow conditions and by Kramer and Valero [22] for higher Froude numbers. The present finding was consistent with the void fraction data, suggesting that the presence of the sidewall had a stronger influence on the development of the shear layer than on the upper recirculating region.

### Bubble chord time and bubble clustering

**Fig. 18:**
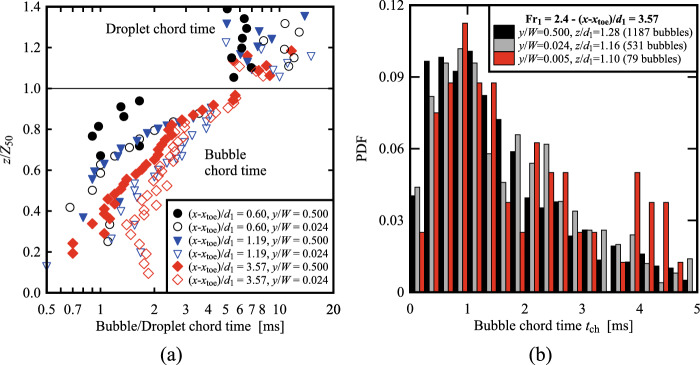
**a** Vertical distributions of the median bubble chord times both in the centreline (*y*/*W* = 0.5) and near the sidewall (*y*/*W* < 0.024) at various locations behind the roller toe, for Fr_1_ = 2.1; **b** PDF of the bubble chord times both in the centreline (*y*/*W* = 0.5) and at multiple locations near the sidewall (*y*/*W* = 0.024 and 0.005) for Fr_1_ = 2.4 at (*x*-*x*_toe_)/*d*_1_ = 3.57

For every bubble/droplet detected by the leading tip of the conductivity probe, its chord time was derived from the air–water signal for *C* < 0.3 (air-bubble) and *C* > 0.7 (water-droplet). The median values for both parameters are presented in Fig. 18a, where the centreline and sidewall data are compared at selected locations behind the roller toe. The data is normalised using *Z*_50_, which is the characteristic elevation where *C* = 0.5, thus implying that values for *z*/*Z*_50_ < 1 represent the bubble chord times, whilst *z*/*Z*_50_ > 1 represent the droplet chord times. The results showed that all locations near the sidewall had a higher median bubble chord time compared to the centreline. More scatter was observed for the droplet chord times in the upper part of the roller, where similar results were observed between the sidewall data and the centreline. The PDFs of the bubble chord times at different locations behind the roller toe for both Froude numbers are presented in Fig. 18b, both in the centreline and near the sidewall. Data showed that at all locations, a lesser number of smaller bubbles was observed near the sidewall (*y*/*W* = 0.024), thus explaining the higher median values observed in Fig. 18a. The data for Fr_1_ = 2.4 at (*x*-*x*_toe_)/*d*_1_ = 3.56 at two locations closer to the sidewalls (*y*/*W* = 0.024 and 0.005) confirmed the same findings. It is important to point out that the distributions near the sidewalls are based on a lesser number of bubbles, which is a consequence of the smaller bubble count rate discussed in Sect. 5.1. These findings confirmed a different dynamic in the shear layer, preventing the smaller bubbles from reaching the sidewall region, possibly linked with the flow features observed in Fig. 6c, d and e.

### Interfacial velocities

**Fig. 19:**
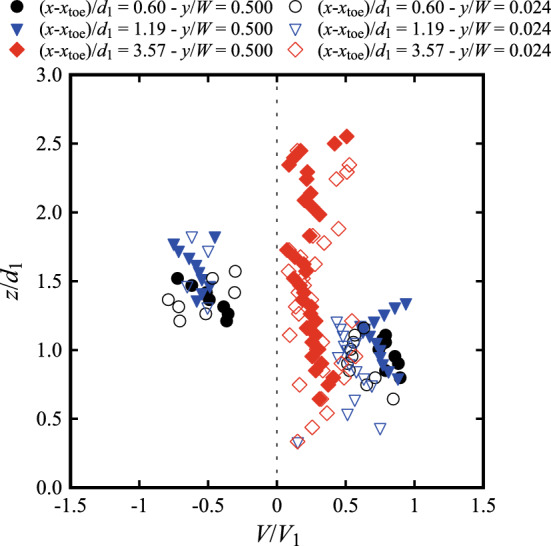
Comparison of the interfacial velocities between the centreline (*y*/*W* = 0.5) and the sidewall (*y*/*W* = 0.024) for Fr_1_ = 2.1. Similar results were obtained for Fr_1_ = 2.4 by Shi et al. [23]

Next to the sidewall, the velocity measurements showed a relative good agreement with the centreline data for Fr_1_ = 2.1 (Fig. 19a). Some slower values were obtained next to the sidewall, especially at the locations closest to the roller toe, *i.e.* (*x*-*x*_toe_)/*d*_1_ = 0.6 and 1.19 (Fig. 19a). The slightly higher scatter observed near the sidewall is associated with the lower presence of air bubbles near the sidewalls, thus affecting the quality of the signal post-processing. Altogether, these data confirmed some preliminary result [29], indicating that the velocities were less affected by the presence of sidewalls than void fractions and bubble-count rate measurements, especially in the downstream part of the roller. Nevertheless, it is acknowledged that the lateral boundary layer development next to the wall was not quantified in details herein. Importantly, the present findings were consistent with stepped chute data, showing lesser velocity magnitude in the close proximity of the sidewalls [21, 24].

### Transverse distributions

**Fig. 20:**
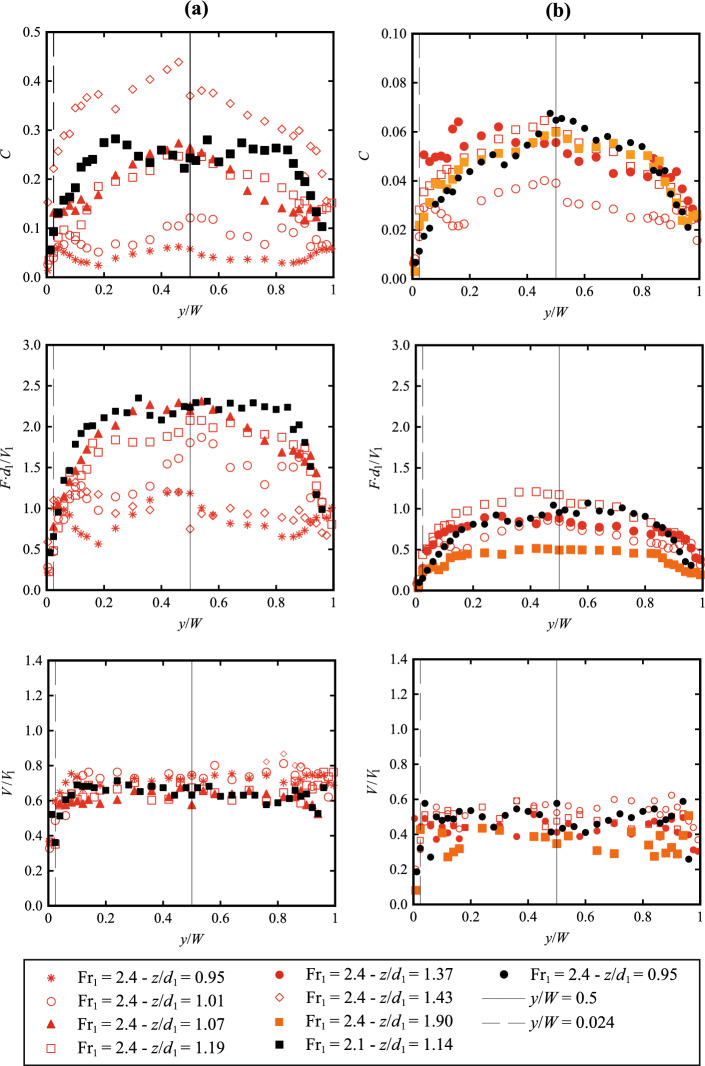
Transverse distributions of air–water properties at selected elevations for: **a** (*x*-*x*_toe_)/*d*_1_ = 1.19 and **b** (*x*-*x*_toe_)/*d*_1_ = 3.57 for Fr_1_ = 2.1 (black symbols) and 2.4 (red/orange). Legend applies to all figures

All measurements next to the sidewall revealed some significant differences in air–water properties compared to the centreline data, hinting the need for greater information on the transverse profiles across the whole channel width. The void fraction and bubble count rate data showed significantly lower values next to the sidewall, compared to the centreline, thus suggesting the presence of transverse patterns across the channel width. At two longitudinal locations within the roller, *i.e.* (*x*-*x*_toe_)/*d*_1_ = 1.19 and 3.57, and at selected vertical elevations (0.95 ≤ *z*/*d*_1_ ≤ 1.90), some complete transverse profiles were recorded for both Froude numbers Fr_1_ = 2.1 and 2.4 (Table 2). The results in terms of void fraction, bubble count rate and interfacial velocities are detailed in Fig. 20, where the black vertical lines mark the two positions (*y*/*W* = 0.024 and 0.5) where the complete vertical profiles of *C*, *F* and *V* were recorded.

Overall, all experimental data revealed a significant variability of the void fraction and bubble/cluster count rate in the transverse direction. At low elevations (*z* ≤ *d*_1_), *C* and *F* presented a somehow undulating transverse profile, suggesting a highly spatial variability within the mixing layer. For *z* > *d*_1_ the transverse profiles presented a parabolic shape, with a maximum value located about the centreline. Although the authors acknowledge the intrusive nature of the phase detection probes, Fig. 20 clearly demonstrates that both void fraction and bubble count rate were drastically smaller next to the sidewall in comparison to the centreline data.

The transverse behaviour of the interfacial velocities measurements across the channel width is presented in Fig. 20. Contrarily to the void fraction and bubble/cluster count rates, velocities presented a lesser variability across the channel width, with a top-hat profile, suggesting a lesser impact of sidewalls away from the sidewall boundary layer region.

### Discussion on transversal distribution of air–water flow properties

This section focused on the air–water flow properties of hydraulic jumps with low Froude numbers next to the sidewall, and on their transverse variations across the full channel breadth. The finding showed conclusively a substantial difference between sidewall and centreline data. This was more marked for the void fraction and the bubble count rate, compared to interfacial velocities. The presence of a transverse pattern across the channel width was identified, confirming the three-dimensional nature of the flow. Although limited to only two Froude numbers and selected locations, the present findings demonstrated that the air–water flow properties next to the sidewalls are not truly representative of the main air–water flow properties in the bulk of the flow. Thus, in the context of today’s race towards more reliable non-intrusive techniques, the present study highlights that image-based measurements through the sidewall would drastically underestimate the rate of air entrainment, the interfacial area and the complicated air–water interfacial interactions as compared to the centreline. This points out the need to understand what exactly sideview optical techniques are capable to detect, leading to a more reliable application of these new, possibly promising approaches.

## Conclusion

The very-strong multiphase turbulence in hydraulic jumps is of practical significance in hydraulic engineering. Although there exists a solid body of literature on hydraulic jumps with high Froude numbers, the jump with low Froude number was rarely studied. Herein, an in-depth understanding of a breaking jump with low Froude number was investigated with various flow conditions and Froude numbers ranging from 2.1 to 2.4. All experiments were conducted in a large-size facility, where intrusive phase-detection probes were used to measure the air–water flow properties, while a ultra-high-speed video camera recorded the flow motion, capturing the fast-evolving nature of the air–water surface features.

Some flow visualisation was first performed for the hydraulic jump with Fr_1_ = 2.1 and 2.4. Both ultra-high-speed-video movies and high-shutter speed dSLR photography highlighted some re-occurring air–water surface features previously observed in breaking bores at the free surface of the roller, including water droplets, fingers, crowns, slugs, spider webs, mushrooms, boils and holes. A qualitative description of these features was documented. These flow features also occurred on the free surface of unsteady breaking bores, thus drawing a similarity of free-surface dynamics between the stationary hydraulic jumps and propagating breaking bores.

The air–water properties were obtained for two hydraulic jumps with low Froude numbers Fr_1_ = 2.1 and 2.4 at relatively high Reynolds number Re ~ 2 × 10^5^. The void fraction followed the air-diffusion and advection process in the breaking roller. The data exhibited three different trends: (1) a monotonic increase near the roller toe with a large gradient was observed near the roller toe; (2) the shear layer formed roughly at (*x*-*x*_toe_)/*d*_1_ > 1, highlighted by a marked peak in the mixing layer; (3) the peak decreased and eventually disappeared further downstream of the roller. Theoretical considerations were given for all three cases of void fraction profiles. The bubble count rate showed a peak in the shear layer for (*x*-*x*_toe_)/*d*_1_ < 2, while two peaks were observed in the shear layer and recirculation zone for (*x*-*x*_toe_)/*d*_1_ > 2. The bubble/droplet chord time data suggested that most bubbles had the longitudinal time scale less than 4 ms, with lower Froude numbers associated with a lower number of bubbles/droplets. Overall, these results for Fr_1_ = 2.1 and 2.4 were in good agreement with previous studies for larger Froude numbers, indicating the applicability of existing theories.

The transverse distributions of air–water flow properties using phase-detection probe showed substantial differences between the sidewall and centreline data. These involved slightly higher free-surface profiles near the sidewalls, larger bubble chord times and values of the void fraction and bubble count rate 2 to 4 times smaller compared to the centreline. On the other hand, there were less differences for the interfacial velocity between the sidewall and centreline, although some boundary effect were observed in the vicinity of the sidewall. These findings point out that image-based results through the sidewall are adversely affected by the sidewall boundary layer effects, and do not represent the air–water flow properties in the centreline. This is an important finding in the current development of optical techniques through sidewalls, pointing out the importance of comparing various independent techniques when assessing the air–water flow properties of turbulent flows.

## Abbreviations

BIV: Bubble image velocimetry
CL: Centreline (*y*/*W* = 0.5)
dSLR: Digital single-lens reflex camera
fps: frames per second
max: Maximum
OF: Optical flow
PIV: Particle image velocimetry
SW: Sidewall (*y*/*W* < 0.024)

## List of symbols

*c*: Instantaneous void fraction
*C*: Time-averaged void fraction
*C*_mean_: Depth-averaged void fraction (Eq. 9)
$C_{{{\text{mean}}}}^{*}$: Depth-averaged void fraction in the roller (Eq. 8)
*d*: Equivalent clear water depth (Eq. 10) [m]
*d*_1_: Upstream water depth [m]
*d*_2_: Downstream water depth [m]
*F*: Bubble count rate [Hz]
*F*_clu_: Cluster count rate defined as the number of clusters per second [Hz]
Fr_1_: Froude number, defined in Eq. (1)
*g*: Gravitational constant [m/s^2^]
*L*_r_: Length of the roller, defined in Eq. (6) [m]
*N*: Exponent in Eqs. 7 and 8
*Ø*: Diameter of the probe [m]
*Q*: Inflow discharge [m^3^/s]
Re: Reynolds number, defined in Eq. (2)
*T*: time of maximum cross-correlation between leading and trailing tip signals [s]
*t*_ch_: Water or air chord time between two bubbles [s]
*V*: Air–water interfacial velocity using phase-detection probe [m/s]
*V*_1_: Inflow flow velocity [m/s]
*W*: Channel width (W = 0.5 m) [m]
*x*: Streamwise coordinate [m]
*x*_toe_: Position of the roller toe, herein *x*_toe_/*d*_1_ = 15.5 [m]
*y*: Transversal coordinate [m]
*z*: Vertical coordinate [m]
*Z*_50_: Vertical position where the void fraction *C* = 0.5 [m]
*Z*_90_: Vertical position where the void fraction *C* = 0.9 [m]
$\Delta x$: Streamwise distance [m] between probe’s tips, herein 5.1 mm < $\Delta x$ < 7.0 mm
$\Delta y$: Transversal separation [m] between two phase-detection probe tips
*μ*: Water dynamic viscosity [kg/m^3^]
*ρ*: Water density [kg/m^3^]
